# Arsenic trioxide (ATO) induced degradation of Cyclin D1 sensitized PD-1/PD-L1 checkpoint inhibitor in oral and esophageal squamous cell carcinoma

**DOI:** 10.7150/jca.47111

**Published:** 2020-09-21

**Authors:** Guanxia Zhu, Xia Li, Jiong Li, Wei Zhou, Zhongjian Chen, Yun Fan, Youhua Jiang, Yue Zhao, Guogui Sun, Weimin Mao

**Affiliations:** 1 Cancer Hospital of University of Chinese Academy of Sciences, Institute of Cancer and Basic Medicine of Chinese Academy of Sciences, Zhejiang Cancer Hospital, Hangzhou, 310022, China.; 2 Wenzhou Medical University, Wenzhou, 325035, China.; 3 Department of Medicinal Chemistry, Massey Cancer Center, Philips Institute for Oral Health Research, Virginia Commonwealth University, Richmond, Virginia 23298-0540, United States.; 4 State Key Laboratory of Molecular Oncology, National Cancer Center/National Clinical Research Center for Cancer/Cancer Hospital, Chinese Academy of Medical Sciences and Peking Union Medical College, Beijing, 100021, China.; 5 North China University of Science and Technology Affiliated People's Hospital, School of Public Health, North China University of Science and Technology, Tangshan, 063001, China.

**Keywords:** esophageal squamous cell carcinoma, oral squamous cell carcinoma, arsenic trioxide, cyclin D1, PD-L1, CDK4/6

## Abstract

Arsenic trioxide (ATO) is widely studied for its antitumor efficacy and several recent studies suggested the immune modulatory effects of ATO in animal models. In this study we found ATO treatment induced increased ROS production and DNA damage in esophageal squamous cell carcinoma (ESCC) cells, led to DNA damage mediated degradation of Cyclin D1 and upregulation of PD-L1 in these cancer cells. Mechanistically, we found ATO induced a transient upregulation and nuclear translocation of Cyclin D1 by sumoylation. Followed with increased ubiquitination and degradation of Cyclin D1 through T286 phosphorylation, and at least partly mediated by Stat1 Y701 phosphorylation. We observed inversed correlations between Cyclin D1 and PD-L1 expression levels in human ESCC tissues. With 4NQO induced PD-L1 humanized mouse oral and esophageal squamous carcinoma model, we found combinatory administration of ATO and check point inhibitor resulted in a significant reduction of tumor volumes. Inversed correlation between Cyclin D1 with PD-L1 was also observed in the 4NQO induced mouse ESCC and OSCC model. Together, these data suggested ATO induced degradation of Cyclin D1 and functional suppression of CDK4/6 pathway sensitized OSCC and ESCC to checkpoint inhibitor treatment.

## Introduction

T cell immunity is an important adaptive cellular immune mechanism that guards the host system against the abnormal cells, including cancer cells. Programmed death ligand-1 (PD-L1) expressed by the cancer cells can bind to programmed death 1 (PD-1) protein on the pro-activated T cells to suppress its activation 1. Checkpoint inhibitors have been proved for the treatments of melanoma, none small cell lung cancer, kidney cancer, hodgkin lymphoma, bladder cancer, oral cell squamous cell carcinoma (OSCC), Merkel cell carcinoma, hepatocellular carcinoma, and cancers with DNA mismatch repair deficiencies or high microsatellite instabilities 2, 3. And FDA recently approved the use of checkpoint inhibitor for the treatment of advanced esophageal squamous cell carcinoma (ESCC) with positive PD-L1 expression 4, 5.

Although checkpoint inhibitors are effective in the treatment of a number of cancers, there are still tremendous needs to increase the efficacies of PD-1/PD-L1 checkpoint inhibitors in cancer treatment 6. Many studies suggested combined usages of checkpoint inhibitors with chemotherapeutic drugs significantly increased the efficacies of checkpoint inhibitors, and have synergistic effects in cancer treatments 7.

Arsenic trioxide (ATO) is known as the first line chemotherapy drug for acute promyelocytic leukemia (APL). Combination use of ATO with all-trans retinoic acid (ATRA) can archive better complete remission rate in the treatment of APL 8. Besides APL, ATO also showed to be effective in treating late stage hepatocellular carcinoma with improved survival rate and objective response rate 9. The State Food and Drug Administration of China approved the use of arsenic trioxide for the treatment of advanced human primary hepatocellular carcinoma (HCC) in 2004 10. Despite of early failures of using ATO as a single agent in the treatment of esophageal cancer, arsenic compounds are still promising to be used as combinatory chemotherapeutic drugs to increase the radiation and chemo sensitivity of solid tumors 11. Several recent studies have highlighted the immune modulatory effects of ATO in animal models. Alex* et al.* reported enhances the NK cell cytotoxicity against acute promyelocytic leukemia. Combination of ATO treatment with NK cell therapy significantly increased the survival time in APL mouse model 12. Wang *et al.* reported the application of ATO as the immune adjuvant in the treatment of mouse hepatocellular carcinoma 13, herein they found ATO significantly improved cytokine-induced killer's cytotoxicity *in vitro* by decreasing CD4^+^ T lymphocytes and Tregs, and increasing CD8^+^ T lymphocytes. In another study, Wang* et al*. reported combination use of ATO with adoptive T cell therapy significantly enhanced cytotoxic T lymphocytes activity, which preferentially decreased the population of Foxp3^+^ Tregs in the tumor centers in the colon cancer lung metastasis mouse model 14. However, the underlying molecular mechanisms for ATO-induced immunomodulatory effects are still not well understood.

CDK4/6 signaling pathway has been reported to modulate the function of PD-1/PD-L1 immune checkpoint 15, 16 and confers cellular resistance to immune checkpoint inhibitors 17. In the present study, we found ATO induced degradation of Cyclin D1 caused a functional suppression of CDK4/6 pathway in ESCC cells. Mechanistically, ATO increased ROS production and DNA damage in ESCC and OSCC cells, which led to a transient upregulation and nuclear translocation of Cyclin D1, followed with DNA damage induced proteasomal degradation of Cyclin D1 at later time points. We further explored the synergistic effects of checkpoint inhibitor with ATO treatment in 4NQO induced mouse oral and esophageal squamous cell carcinoma model. ATO Treatment significantly increased efficacy of checkpoint inhibitor in mouse oral squamous cell carcinoma (OSCC) and esophageal squamous cell carcinoma (ESCC).

## Materials and Methods

### Cancer cell lines and cancer tissues

ESCC cell lines KYSE150, KYSE450 were preserved in our laboratory. The ESCC cells were cultured in RPMI 1640 (Gibco) supplemented with 10% fetal bovine serum (FBS) at 37°C under 5% CO2. OSCC cell line (SSC1), kindly provided by Professor Demeng Chen of Cancer Hospital of Sun Yat-sen University, was cultured in Dulbecco's modified Eagle's medium (DMEM) containing 10% FBS at 37°C under 5% CO2.

ESCC cancer tissues were collected from the cancer tissue bank of Zhejiang Cancer Hospital, of which were derived from 97 patients (median age, 64; range, 51-84; 81 men and 16 women) diagnosed with ESCC who had undergone curative surgery at Zhejiang Cancer Hospital (Hangzhou, China) in 2015. After being checked by the pathologist, the tumor tissues and paired normal esophageal tissue were immediately frozen in liquid nitrogen and stored at -80°C for further research. All tissue specimens used in our study were obtained from the tissue bank of Zhejiang Cancer Hospital and all patients were informed and consent with the study before surgery. This study was approved by the Institutional Ethical Review Board of Zhejiang Cancer Hospital.

### DCFDA ROS detection assay

ESCC KYSE-150 cells were seeded on Millicell EZ slides, and stained with 25µM of DCFDA purchased from Sigma (D6883) for 40 mins. Then the cells were washed with PBS for 3 times, the cells were cultured back in 1640 full growth media and treated with ATO for 12 hours before fluorescence microscope observation with excitation/emission at 495 nm/529 nm.

### Single cell gel electrophoresis (comet) assay

The comet assay was performed with DNA damage Assay kit (Nanjing Jiancheng bioengineering institute G010-1-1) following the manufacture protocol. Briefly, KYSE-150 cells were treated with ATO for 24 hours. After ATO treatment, the medium was removed and the cells were washed with PBS, the cells were harvested, and resuspended in PBS at a cell density of 1×106. The cells were mixed with molten LM Agarose (42°C) at a ratio of 1:8 (v/v), and 85μl of the mixed solution was immediately pipetted onto a glass slide. The slides were placed flat at 4°C for 30 min and then immersed in lysis solution on ice for 1 hr. The lysis buffer was removed, and the slides were immersed in alkaline solution (1 mmol/L EDTA and 300 mmol/L NaOH, pH > 13) for 1 hr. Slides were washed three times for 10 min with pre-chilled Neutralization buffer (0.4 mmol/L Tris-HCl) after electrophoresed at 1 volt/cm (25 v) for 20 min. Slides were stained with propidim iodide for 10 min. The slides were viewed with fluorescence microscope (Olympus FV-1200 confocal) and analyzed using the Comet Assay Software Project (CASP).

### Luciferase reporter assay

pGME2F-Luciferase reporter plasmid was purchased from Yeasen Biotechnology Company, the construct was transfected into KYSE-150 cells with Lipofectamine 3000®. 24 h after transfection, the KYSE-150 cells were treated with ATO (10 µM) for 48 hours. Then the cell lysates were collected, and luciferase activity was measured using a Yeasen Luciferase reporter gene assay kit (11401ES60).

### Cell cycle analysis

Cellular DNA content was measured by flow cytometric analysis after 48 h incubation of KYSE150 cells with ATO (10 µM). Briefly, 1 × 10^6^ cells from control (untreated) and treated cells were washed twice with cold PBS, and then fixed in 70% ethanol at 4°C overnight. The cells were then stained with propidim iodide (50 µg/ml) at 37°C for 30 min. The samples were analyzed with a FACSC antoTM Flow Cytometry System (BD Biosciences). And the data were analyzed using FlowJo VX software according to the manufacturer's instruction.

### Realtime PCR

For the real-time PCR measurement of cyclin D1 RNA levels in the ESCC cells, approximately 10^6^ of KYSE-150 Cells were harvested from the Petri dish after ATO (10 µM) treatment for 48 hours with a cell scrape stick, and suspended in 1ml PBS. The cell suspensions were briefly centrifuged, and the pellet were resuspended in 1 ml Trizol (Invitrogen). For the real-time PCR measurement of cyclin D1 mRNA levels in the human tissue samples, 97 cases of human ESCC tissue samples with paired normal esophageal tissues were randomly picked for real-time PCR assay. Then the Cell suspensions Tissue samples were immediately posited in 1 ml Trizol (Invitrogen) Reagent after taking out from -80°C refrigerator, and further processed with TissueLyser for RNA extraction. Total cellular RNA was extracted by the Trizol Reagent according to the supplier's instructions. cDNAs of the samples were synthesized with RevertAid First Strand cDNA synthesis Kit (Thermo Fisher). And real-time PCR amplification of the cDNAs were performed with SYBR® Premix Ex Taq™ (TAKARA) kit in ABI 7500 real-time PCR system, with the following specific primers in table 1.

The PCR conditions included a denaturation step at 95°C for 5 min, followed by 35 cycles of denaturation at 95°C for 10 s, annealing at 58°C for 15 s, and extension at 72°C for 10 s. Detection of the fluorescent product was carried out at the end of the 72°C extension period. Each sample was tested at least in triplicate and repeated using three independent cell preparations.

### Isolation of cytoplasmic and nuclear proteins

Nuclear/cytosol protein extraction was performed with Solarbio nuclear protein extraction kit (R0050). Briefly, the KYSE-150 cells were harvested and washed with phosphate-buffered saline (PBS), and then centrifuged at 500 × g for 5 min. After removal of the PBS, the cell pellet was resuspended with 100 µL of nuclear and cytoplasmic extraction reagent (140 mM NaCl, 1.5 mM MgCl_2_, 10 mM Tris-HCl pH 8.5, 0.5% NP-40), incubated on ice for 5 min, and centrifuged at 5000 × g for another 5 min. The supernatant was then removed to a new tube, and 1 mL Trizol reagent was added (cytosol fraction). After washing with PBS, the nuclear pellet was also resuspended in 1 mL of Trizol reagent. Then the nuclear and cytosol fractions of proteins were further extracted from the Trizol solution according to the kit protocol.

### Western blotting, immunoprecipitation and ubiquitination assay

Western blot experiments were performed with cell lysates harvested at different time points after ATO treatment. Cells were lysed in PierceTM IP lysis buffer (Thermo). Protein loading buffer for electrophoresis is composed with (8 M urea, 1 M thiourea, 0.5% 3-((3-cholamidopropyl) dimethylammonium)-1- propanesulfonate (CHAPS), 50 mM dithiothreitol (DTT) and 24 mM Spermine). 2 µl anti-cyclin D1 mAb (Abcam), 20 µl of 50% slurry of protein G beads were added to each samples incubated at 4°C for 16hours at a rotator in the immunoprecipitation experiment. Beads were washed four times with 1 ml of 10-fold diluted urea buffer and 20 µl of 2 × SDS loading buffer was added. Immunoprecipitations were analyzed by western blot as indicated. Antibodie: anti-cyclin D1 antibody (Abcam), anti-γH2AX antibody (Santa cruz), anti-Cul3 (Proteintech), anti-PDL1 (Proteintech), anti-β-actin (Proteintech), anti-Lamin B1 (Proteintech), anti-pSTAT1 (CST), anti-pCyclin D1 (T286) (CST). Sumoylated cyclin D1 and ubiquitylated cyclin D1 was detected by co-immunoprecipitation using anti-SUMO-2/3 antibody or anti-ubiquitin antibody conjugated beads (Enzo Life Science), followed by immunoblotting with anti-cyclin D1 antibody or for cyclin D1 detection.

### Immunohistochemistry experiment

Haematoxylin and eosin staining were performed to confirm the diagnosis, and analysis of pathological grades. All the tissues were fixed in 4% neutralized formaldehyde, embedded in paraffin. Blocks of paraffin-embedded donor tissue were sampled using a Manual Tissue Arrayer 1 instrument (Beecher Instruments, Silver Spring, MA, USA). Formalin-fixed paraffin-embedded (FFPE) tumor tissue samples were hematoxylin and eosin (H&E) stained, and ESCC was confirmed by pathologist. The paraffin tissue blocks of 97 ESCC cases with paired normal esophageal tissues were used in the construction of tissue microarray. In brief, the H&E-stained standard slides were reviewed from each section of esophageal cancer tissues and one representative tumor area of each tumor (1.5 mm diameter) were removed from FFPE tissue blocks. A serial of 4-μm-thick sections were cut for the purpose of immunohistochemistry and transferred to adhesive slides according to manufacturer's instructions.

Standard IHC analysis was performed with the primary antibodies against human Cyclin D1 (CST (92G2) Rabbit mAb #2978), Cul3 (Proteintech 11107-1-AP), Stat1-p (CST Phospho-Stat1 (Tyr701) (58D6)), PD-L1 (Proteintech 66248-1-Ig) at a dilution in 1:100. In brief, the tissue microarray slides were deparaffinized in xylene and gradient ethanol. Antigen retrieval was performed by placing the slides in a high-pressure cooker in a 0.01 mM citrate buffer, pH 6.0, for 2.5 min at 100°C; they were then cooled for 20 min. Endogenous peroxidase activity was blocked by incubating the section in 3% H2O2 for 10 min, followed by rinsing in PBS solution three times, tissue microarray slides were preincubated with blocking serum and then were incubated with the primary antibodies at 4°C overnight. After three washes in PBS, the slides were treated with the horseradish peroxidase (HRP)-labeled goat anti-mouse/ rabbit secondary antibody (Dako, Glostrup, Denmark) for 50 minutes at room temperature. After washing with PBS, reaction products were visualized with 3, 3′-diaminobenzidine (DAB, Dako, Glostrup, Denmark) and the slides were counterstained with hematoxylin. After being dehydrated, slides were mounted in resin. Immunohistochemistry results were evaluated by scanning each slide under low power magnification (×100) to identify regions containing positive immunoreactivity.

### Immunofluorescence microscopy

KYSE-150 cells grown on Millicell EZ slides (EMD Millipore) were fixed in 4% PBS paraformaldeyde for 20 min, incubated in 0.3% Triton-X-100 for 15 min. After blocking with IF blocking buffer, the slides were stained with an anti-cyclin D1 antibody (Abcam), anti-γH2AX antibody (Santa cruz) overnight at 4°C. Antibodies were used at 1: 200 dilution in PBS. The cells were stained with the secondary antibody for 60 min and DAPI (biosharp, Hefei, China) for 10 min at room temperature away from light and EZ slides were visualized by a fluorescence microscope (Olympus FV-1200 confocal).

### Mouse tumor implantation

Murine Hepa1-6 cells were purchased from Sciencell. Hepa1-6 cells were cultured in DMEM medium. The Hepa1-6 tumor models were generated by subcutaneous injection of 2×10^6^ cells into the flank of C57/B6 mice, purchased from Hangzhou Sike Biotech. Mice were housed in a standard experimental animal facility according to the national laws and policies. When the tumor volume reached approximately 100 mm^3^, the mice were randomized and intraperitoneally administrated with ATO (2 mg/kg) or PBS every other day for 2 weeks. The tumors were excavated and lysed for Western Blot analysis when reached approximated 2000 mm^3^.

### 4NQO induced mouse OSCC and ESCC model

Humanized C57BL/6J-Cd274^em2(hPD-L1)Smoc^ mice, purchased from Shanghai Model Organisms, were fed with 4NQO water (100 µg/ml) for 16 weeks starting at six weeks of age. 4NQO was withdrawn from the the drinking water of the mice after 16 weeks, and the mice were further kept for 4 weeks. Mice were grouped into nontreatment control group, ATO treatment group, checkpoint inhibitor Durvalumab treatment group, and ATO plus Durvalumab treatment group. Checkpoint inhibitor was delivered by intraperitoneal administration of Durvalumab (200 µg/mice in 200 µl PBS) every three days for 8 times. ATO treatment was delivered by intraperitoneal injection 2 mg/kg every other day for 3 weeks. By the end of the treatment period, mice were sacrificed, the anatomized and tumors of the oral cavity and esophagus were and measured. Afterward, the tumors were dissected and put into formaldehyde and prepped for IHC staining. ATO used in this study was purchased from Beijing Puxi Scientific. Checkpoint inhibitor Durvalumab was provided by AstraZeneca.

## Results

### ATO induced G2/M cell cycle arrest and degradation of Cyclin D1 in cancer cells

**Figure 1:**
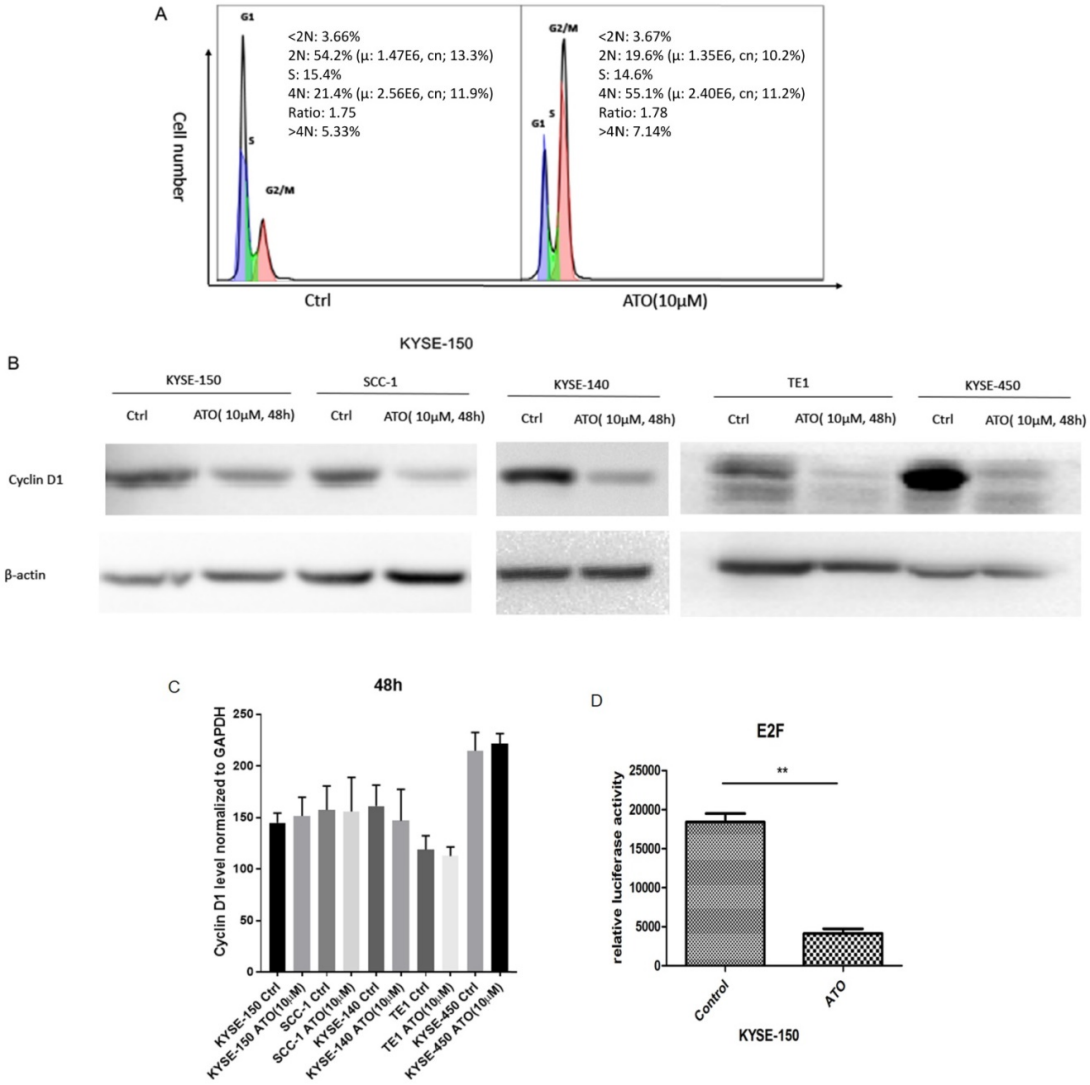
(**A**) Flowcytometry analysis showed 48h of ATO (10µM) treatment in ESCC KYSE-150 cells induced G2/M cell cycle arrest. (**B**) 48 h of ATO treatment of ESCC cancer KYSE-150, KYSE-140, TE1, KYSE-450 and OSCC SCC-1 cells induced downregulation of Cyclin D1 protein levels by Western Blot. (**C**) The mRNA levels of Cyclin D1 is measured with Real-time PCR assay in KYSE-150, SCC-1, KYSE-140, TE1, KYSE-450 cells 48 hours after ATO treatment. (**D**) E2F luciferase reporter assay showed a significant downregulation of E2F reporter activity by ATO treatment in KYSE-150 cells for 48 hours.

Previous studies showed ATO treatment induced G1/S and G2/M cell cycle arrests in cancer cells 18-21. We found ATO induced G2/M cell cycle arrest in ESCC KYSE-150 Cells (Figure 1A). Cyclin D1 depletion is known to cause both G1/S and G2/M cell cycle arrests, which is regulated by the cyclin D1-CDK4/6 and cyclin D1-Chk1-Cdc25-Cdc2 pathway under oxidative stress respectively 22, 23. We speculated that cyclin D1 might be a molecular target ATO treatment in ESCC cells. With western blot assay, we found ESCC and OSCC cell lines treated with ATO (10 µM) for 48 hours robustly induced cyclin D1 degradation (Figure 1B). To test if ATO treatment repressed cyclin D1 transcription, we measured cyclin D1 RNA levels with real-time PCR, and found no significant changes of cyclin D1 mRNA levels in ESCC and OSCC cells by ATO treatment (Figure 1C). With E2F luciferase reporter assay, we found ATO treatment induced significant suppression of CDK4/6 signaling pathway, which suggested ATO induced cyclin D1 degradation and caused functional suppression of the CDK4/6 signaling pathway (Figure 1D).

### ATO induced Cyclin D1 degradation is mediated by DNA damage response

**Figure 2:**
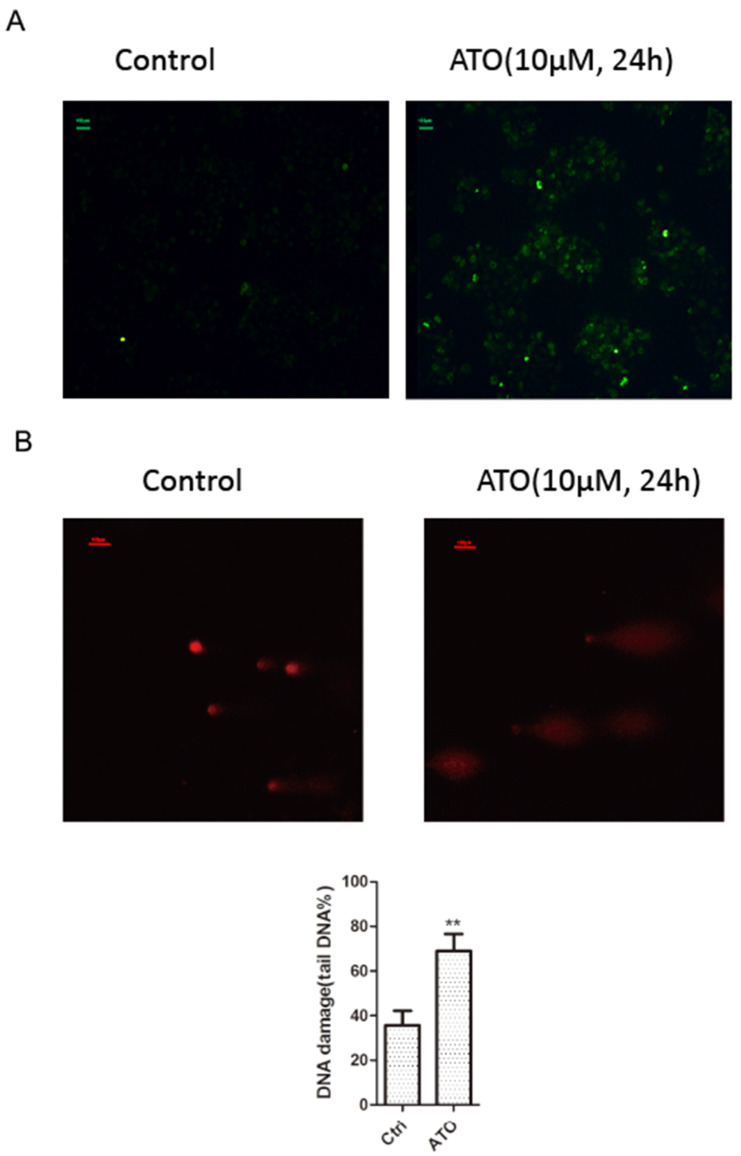
(**A**) Fluorescent microscope observation indicated a significant increase of 2′, 7′-Dichlorofluorescin (green) upon 24 h of ATO treatment in KYSE-150 cells. (**B**) Fluorescent microscope observation of single cell comet assay of DNA damage indicated a significant increase of DNA double strand breaks upon 24 h of ATO treatment in KYSE-150 cells, the red fluorescent of DNA is stained by PI (propidium iodide).

**Figure 3:**
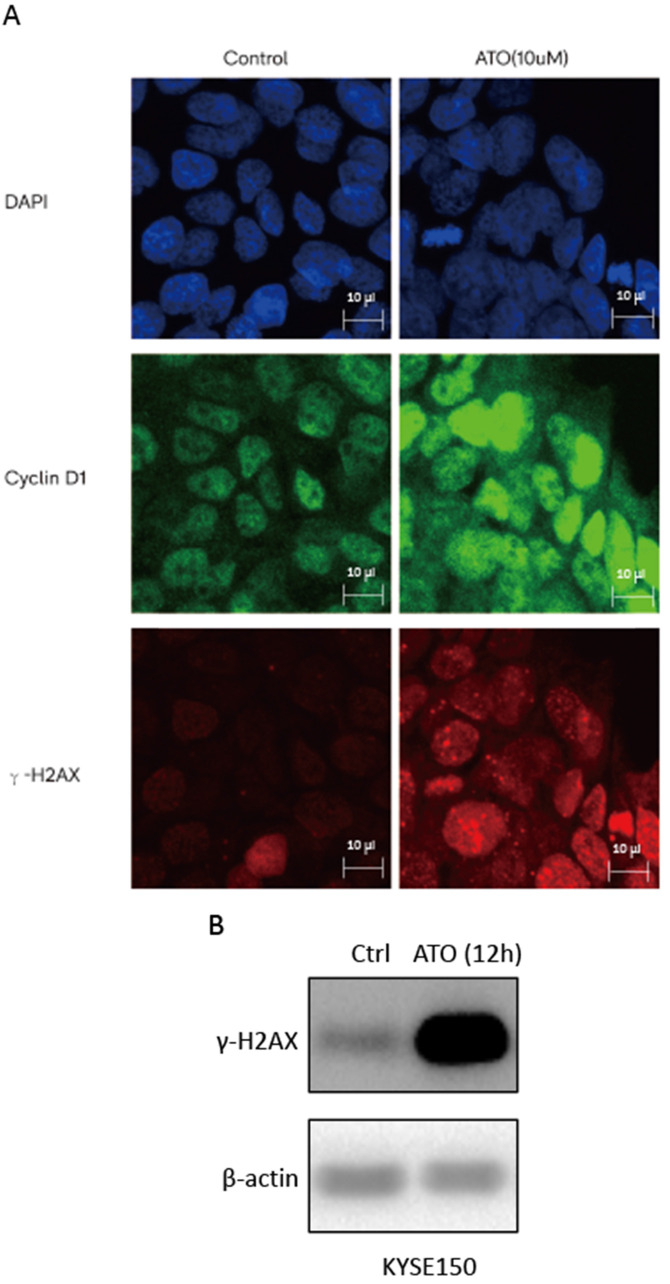
(**A**) Confocal immunofluorescent microscopy images indicated a transient upregulation and nuclear translocation of Cyclin D1 and a significant increase of γH2AX foci upon 6 hours of ATO treatment. (**B**) Western Blot showed a significant upregulation of γH2AX upon 12 hours of ATO treatment in KYSE-150 cells.

ATO is a potent inducer of reactive oxygen species (ROS), which can cause DNA damages in cancer cells 24, 25. With cellular 2′,7′-Dichlorofluorescin diacetate (DCFDA) ROS detection assay, we observed a significant increase of ROS production in ESCC KYSE-150 cells after ATO treatment (Figure 2A). With DNA damage comet assay, we observed a significant increase of DNA double stand breaks after ATO treatment in KYSE-150 cells (Figure 2B). We observed a significant increase of γH2AX foci in the nucleus of KYSE-150 cells after ATO treatment for 12 h with confocal immune fluorescent microscope. (Figure 3A) We also observed an increase of γH2AX protein level after ATO treatment in KYSE-150 cells by Western blot (Figure 3B).

**Figure 4:**
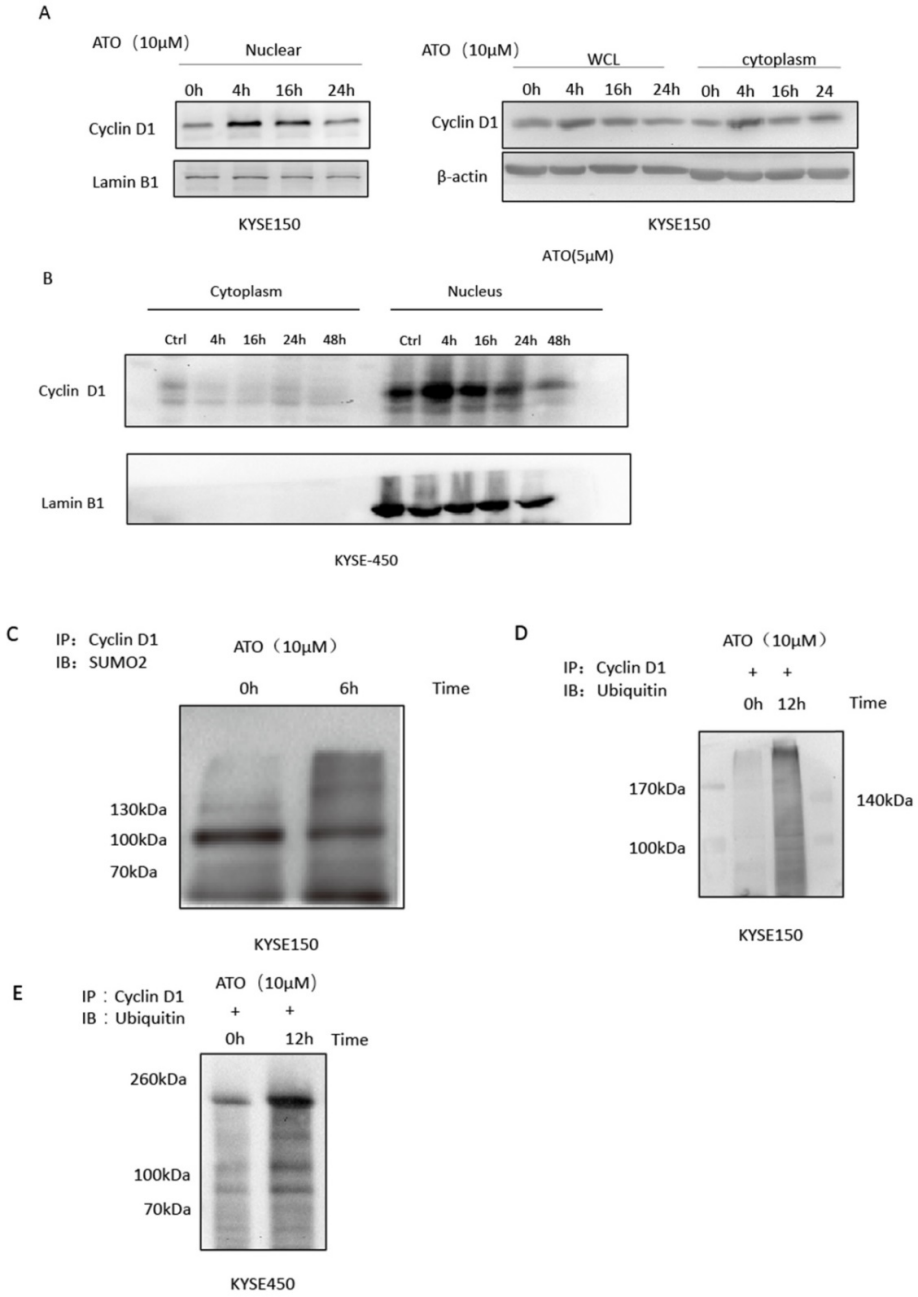
(**A**) Comparation of Cyclin D1 protein levels of the nuclear and cytoplasmic faction at different timepoints after ATO treatment by western blot indicated a transient upregulation of Cyclin D1 prior to its degradation upon ATO treatment in KYSE-150 cells, particularly in the nuclear fraction of the cells. (**B**) Comparation of Cyclin D1 protein levels of the nuclear and cytoplasmic faction at different timepoints after ATO treatment with KYSE-450 by western blot indicated a transient upregulation of Cyclin D1 prior to its degradation upon ATO treatment in the nuclear fraction of the cells. (**C**) A significant increase of Sumoylated Cyclin D1 was observed by immunoprecipitation assay upon ATO treatment. (**D**) A significant increase of ubiquitinated Cyclin D1 upon ATO treatment in KYSE-150 cells was observed by immunoprecipitation assay. (**E**) A significant increase of ubiquitinated Cyclin D1 upon ATO treatment in KYSE-450 cells was observed by immunoprecipitation assay.

Cyclin D1 is involved in DNA damage repair by recruiting Rad51 to the DNA repair sites to promote homologous recombination mediated repair 26. By comparing cyclin D1 protein levels of the nuclear and cytosol fractions of the cells at different time points after ATO treatment, we found ATO induced nuclear accumulation of cyclin D1 prior to its degradation in ESCC KYSE-150 and KYSE-450 cells (Figure 4A, B). We also observed a transient upregulation of Cyclin D1 protein level, and increased of nuclear translocation of cyclin D1 upon ATO treatment in KYSE-150 cells with confocal immunofluorescent microscopy (Figure 3A). Wang* et al.* reported Sumo modified cyclin D1 primarily resided in the cell nucleus, and sumoylation of cyclin D1 is important for its nuclear translocation and oncogenic functions 26. We observed increased sumoylated cyclin D1 in KYSE-150 cells by ATO treatment (Figure 4C). Thus, ATO induced sumoylation of cyclin D1 might be the underlying causes for the nuclear translocation and the transient upregulation of cyclin D1 by ATO treatment in KYSE-150 cells. Increased ubiquitinated cyclin D1 is also observed in KYSE-150 and KYSE-450 cells upon ATO treatment, suggesting ATO induced cyclin D1 degradation is mediated by the ubiquitination mediated proteasomal degradation pathway (Figure 4D, E).

**Figure 5:**
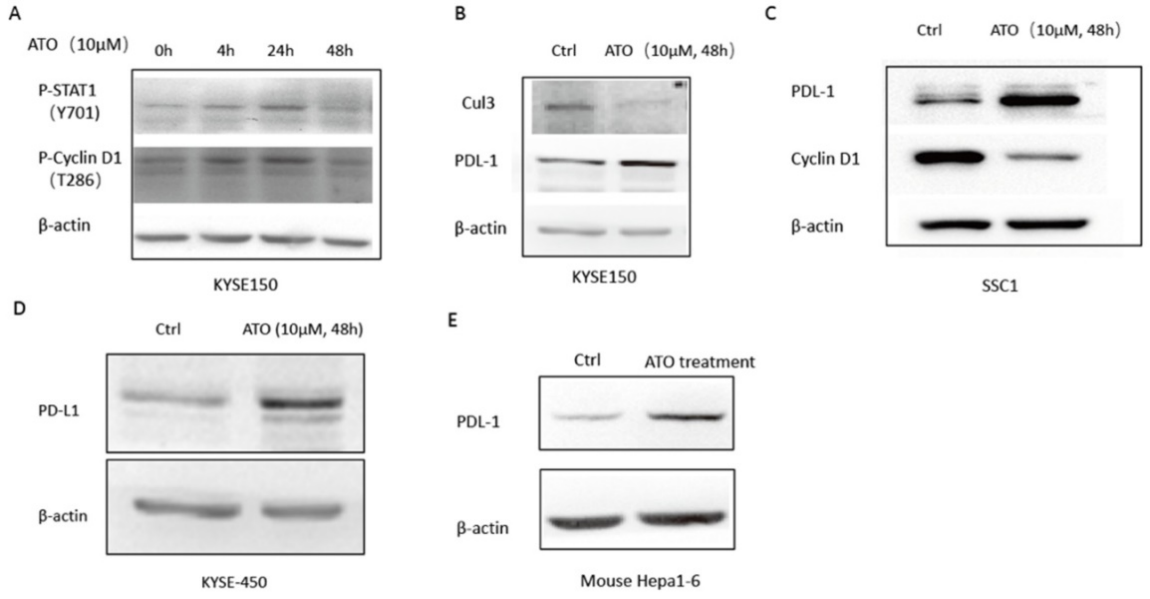
(**A**) 48 hours of ATO (10 µm) treatment of KYSE-150 cells induced transient upregulations phosphorylation of T286 site of Cyclin D1 AND Y701 site of Stat1 as indicated by Western blot assay. (**B**) 48 hours of ATO (10 µm) treatment induced downregulation of Cul3 and upregulation of PD-L1 in ESCC KYSE-150 cells. (**C**) ATO (10 µm) treatment induced downregulation of Cyclin D1 and upregulation of PD-L1 in OSCC SCC1 cells was observed by Western blot. (**D**) 48 hours of ATO (10 µm) treatment induced upregulation of PD-L1 in KYSE-450 cells was observed by Western blot. (**E**) ATO (10 µm) treatment induced PD-L1 upregulation in Hepa1-6 mouse hepatocellular carcinoma cells in the mouse xenograft model was observed by Western blot.

DNA damage induced T286 phosphorylation of cyclin D1 by GSK3β has been reported to mediate the ubiquitination and degradation of cyclin D1 27, 28. Wang *et al.* reported ATO activated GSK3β by inhibiting ERK/AKT signaling in APL NB4 cells 9. We also observed ATO treatment increased T286 phosphorylated cyclin D1 in KYSE-150 cells (Figure 5A), which suggested ATO induced DNA damage promoted proteasomal degradation of Cyclin D1 by T286 phosphorylation. Additionally, Dimco *et al*. reported phosphorylated Stat1 directly interacted with cyclin D1 to promote its proteasomal degradation 29. We also observed increased phosphorylated Stat1 (Tyr701p) by ATO treatment in ESCC KYSE-150 cells (Figure 5A).

### ATO induced Cyclin D1 degradation causes upregulation of PD-L1

Cyclin D1-CDK4/6 pathway is known to regulate PD-L1 stability through transcriptional induction of Cul3-Spop E3 ligase, which mediates the degradation of PD-L1 15. We observed down regulation of Cul3, and upregulation of PD-L1 by ATO treatment in ESCC KYSE-150 (Figure 5B). Consistently, ATO induced downregulation of Cyclin D1 and upregulation of PD-L1 were also observed in OSCC SCC1 cells and ESCC KYSE-450 cells (Figure 5C, D). Consistently, mouse xenograft model with mouse hepatocellular carcinoma cell line Hepa1-6 cells also showed with upregulation of PD-L1 after 10 consecutive intraperitoneal treatments of ATO (Figure 5E).

### Immunohistochemistry analysis of Cyclin D1, Stat1 and PD-L1 in human ESCC tissue samples

**Figure 6:**
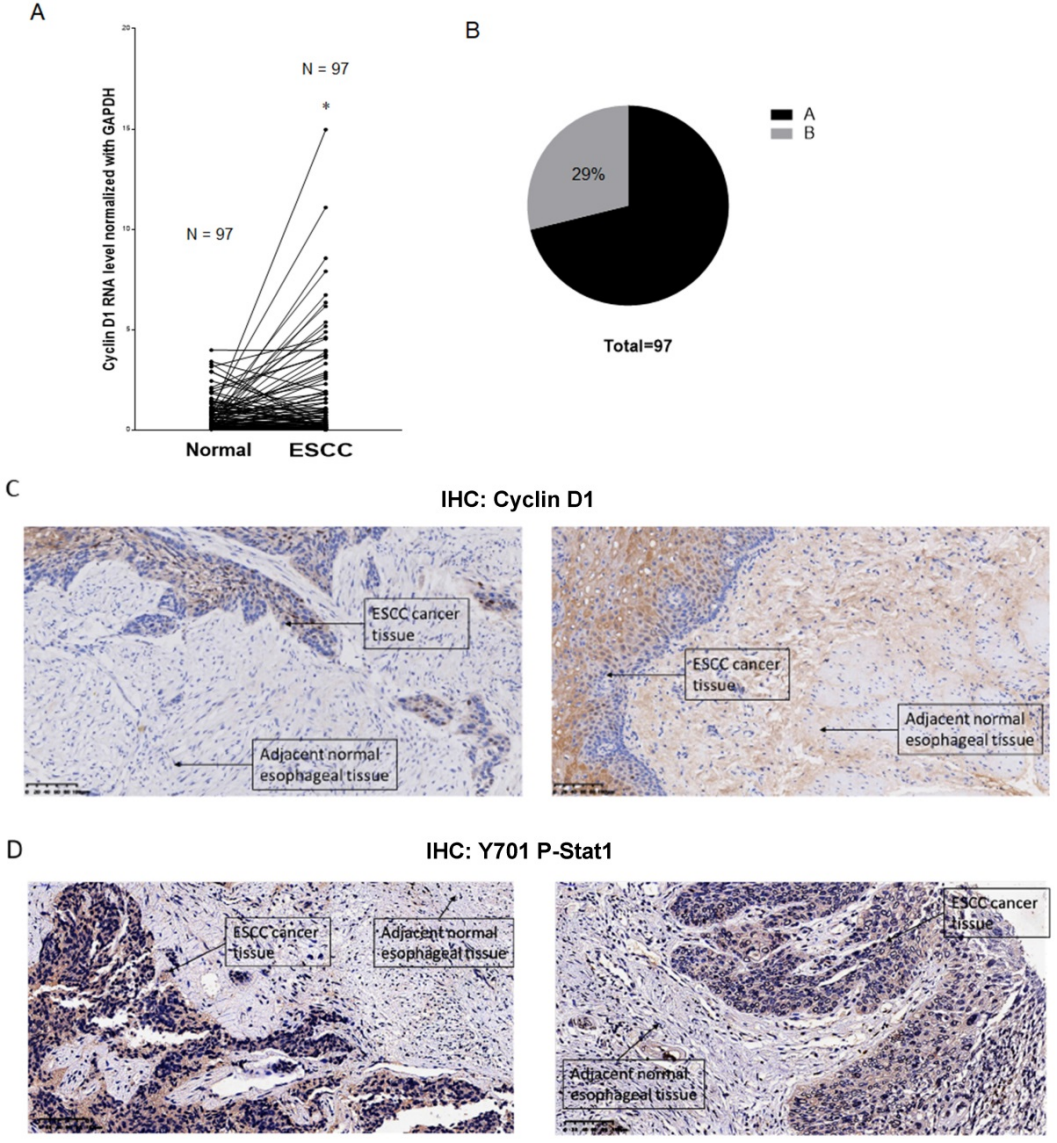
(**A**) Paired analysis of Cyclin D1 mRNA levels of human ESCC tissues with adjacent normal esophageal tissues by realtime PCR showed Cyclin D1 mRNA levels were upregulated in 63% of the ESCC tissues. (**B**) Paired analysis of Cyclin D1 protein levels of human ESCC tissues with adjacent normal esophageal tissues by immunohistochemistry (IHC) showed Cyclin D1 protein levels were upregulated in 29% of the ESCC tissues. (**C**) IHC photos of human ESCC tissues showed with significant upregulations of Cyclin D1 in ESCC cancer tissues compared with adjacent normal esophageal tissues, the darker brown color was stained by DAB catalyzed by HRP labeled secondary antibody against Cyclin D1 primary antibody (Abcam), the nucleus were stained by DAPI. (**D**) IHC photos of human ESCC tissues showed with significant upregulations of Y701 P-Stat1 in ESCC cancer tissues compared with adjacent normal esophageal tissues, the darker brown color was stained by DAB catalyzed by HRP labeled secondary antibody against Y701 P-Stat1 primary antibody (CST), the nucleus was stained by DAPI.

**Figure 7:**
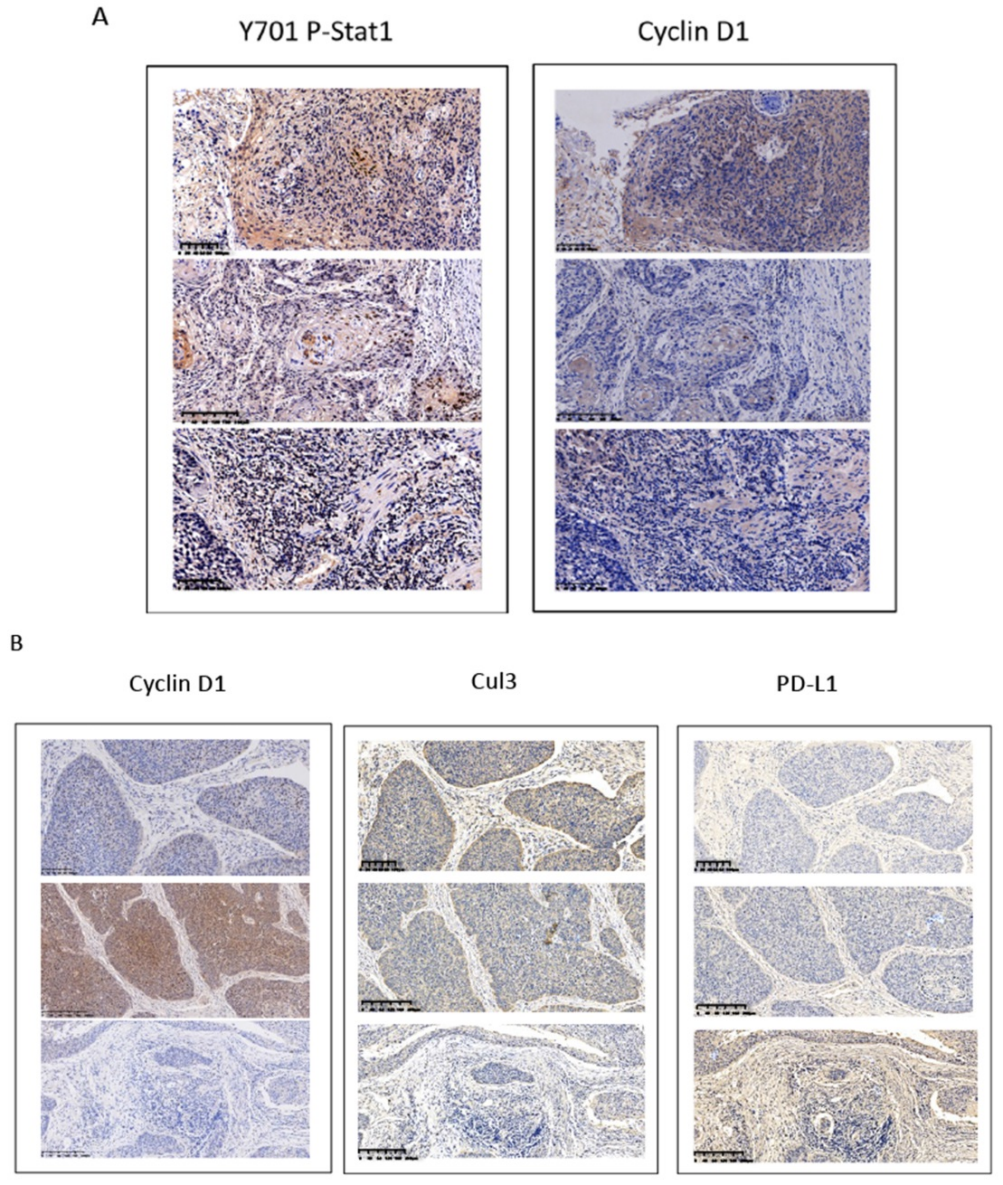
(**A**) Inversed correlations between Y701 P-Stat1 and Cyclin D1 levels are found in ESCC tissue samples with IHC staining. (**B**) Inversed correlations between Cyclin D1 and Cul3 with PD-L1 levels were shown in human ESCC tissues with IHC staining.

Overexpression of cyclin D1 is found in a large number of cancers, including esophageal cancers, head and neck cancers, breast cancers, lung cancers etc. 30. Previous cancer genetic study indicated cyclin D1 gene is amplified in 46.4% of the ESCC among the Chinese population 31. Realtime PCR results indicated upregulation of cyclin D1 RNA indicated cyclin D1 RNA is upregulated in 63% of ESCC tissue samples (Figure 6A). However, with IHC assay we observed Cyclin D1 is overexpression in only 39% of the human ESCC tissues samples (Figure 6B, C), which is significantly lower compared with cyclin D1 RNA upregulation in ESCC tissues. Zhang* et al.* reported 49% of cases of human ESCC tissue samples showed with a strong positivity of Stat1 in immunohistochemistry analysis, and ESCC patients with strong Stat1 positive scores in the IHC analysis survived significantly longer than those with STAT1-weak/negative tumors 32. We also observed Tyr701 phospho-Stat1 is upregulated in a significant proportion of ESCC cancer samples (Figure 6D). And the positivity of phospho-Stat1 staining is inversely correlated with the positivity of cyclin D1 staining in ESCC patient tissues (Figure 7A). Activated Stat1 have been reported to directly interact with cyclin D1 to promote its proteasomal degradation in fibrosarcoma cancer cells 29. Together, these data suggested upregulation of p-Stat1 (Y701) in ESCC tissue samples may cause an increase of proteasomal degradation of cyclin D1, resulted in a less dramatic upregulation ratio of cyclin D1 protein levels in ESCC tissues. With IHC analysis we also observed the expression levels of PD-L1 were inversely correlated with the protein levels of cyclin D1, Cul3 in human ESCC tissue samples (Figure 7B).

### ATO has a synergistic effect with checkpoint inhibitor in cancer treatment with 4NQO induced squamous cell carcinoma mouse model

**Figure 8:**
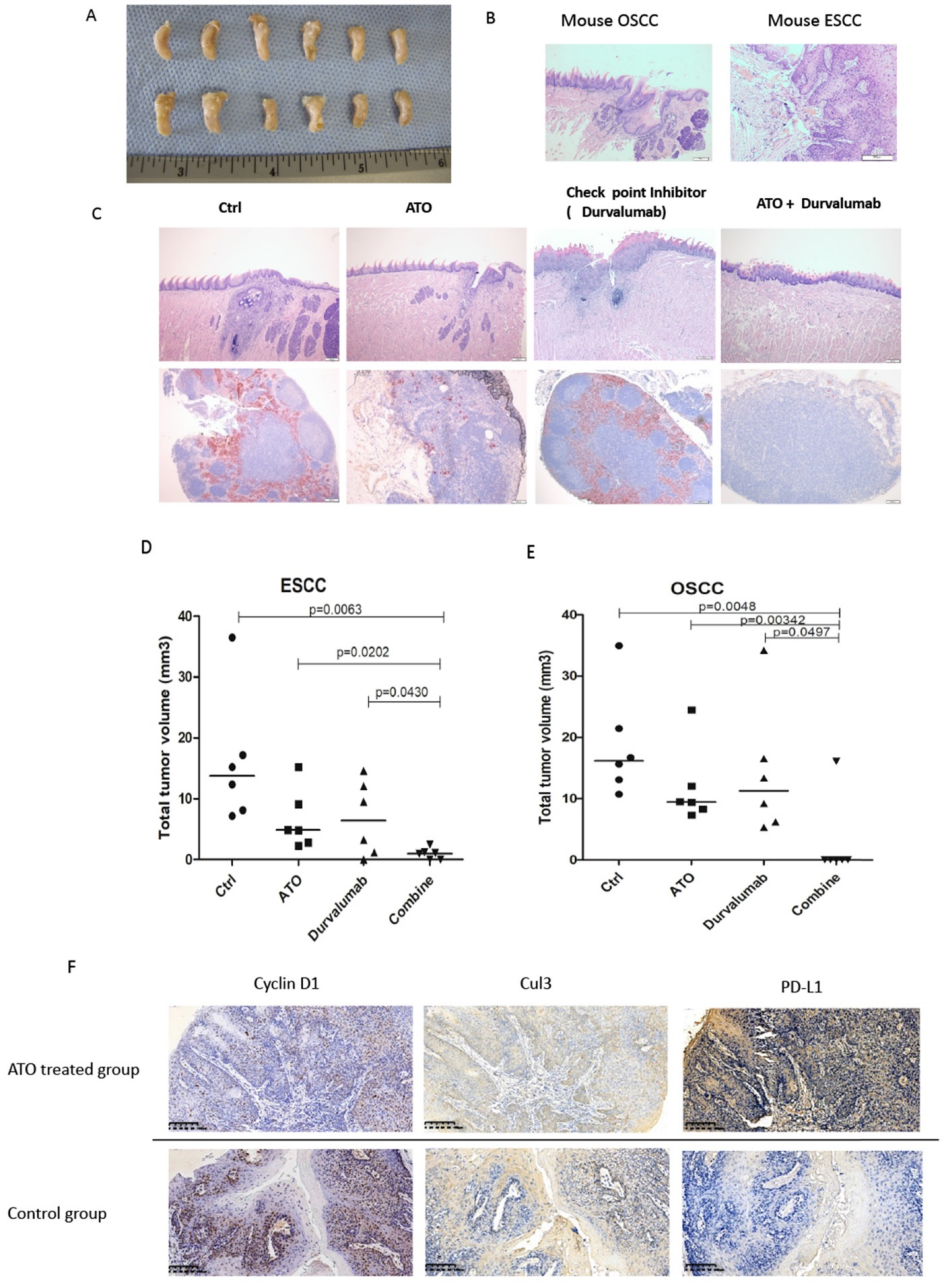
(**A**) At week 24, PD-L1 humanized C57 mice were sacrificed, the tong and esophagus were anatomized, OSCC tumor tissues and ESCC tumor tissues over the tongue and esophagus were dissected, fixed with Formalin and embedded with paraffin, and processed for IHC analysis. (**B**) Scanned pictures showed with the pathological features of mice OSCC and ESCC tissues by HE staining in the IHC analysis. (**C**) Photos of OSCC HE staining of PD-L1 humanized mice from different treatment group. (**D**) Scattered dots illustration of tumor sizes of 4NQO induced mouse ESCC model, 6 mice were included in each group, the mice were treated with ATO, checkpoint inhibitor (Durvalumab), and combination of ATO and Durvalumab. (**E**) Scattered dots illustration of tumor sizes of 4NQO induced mouse OSCC model, 6 mice were included in each group, the mice were treated with ATO, checkpoint inhibitor (Durvalumab), and combination of ATO and Durvalumab. (**F**) Inversed correlations were shown between Cyclin D1 (and Cul3) with PD-L1 levels in 4NQO induced mice ESCC tissue samples with IHC staining.

4NQO (4-Nitroquinoline 1-oxide) is a potent inducer of oral and esophageal squamous cell carcinomas (SCC) 33. We used 4NQO induced mouse SCC model to study the synthetic effect of ATO plus checkpoint inhibitor treatment in PD-L1 humanized C57 mice (C57BL/6J-Cd274**^em2(hPD-L1)Smoc^**). By adding 4NQO into the drinking water for 16 weeks, PD-L1 humanized mice developed with oral and esophageal cancers. At week 20, PD-L1 humanized mice were treated with ATO plus PD-1/PD-L1 checkpoint inhibitor (Durvalumab), compared with ATO or Durvalumab treatment alone, the mice were sacrificed at week 24. With anatomical and immunohistochemistry analysis we found significantly smaller sizes of ESCC and OSCC in the ATO and checkpoint inhibitor combinatory treatment group of mice (Figure 8A-C).

Immunohistochemistry analysis of the mouse OSCC and ESCC tissues of the 4NOQ induced OSCC/ESCC model showed downregulations of cyclin D1, Cul3 protein levels in the ATO treated groups. We also observed inversed correlations between the protein levels of cyclin D1/Cul3 and PD-L1 with IHC analysis in the 4NQO induced mouse SCC model (Figure 8C). Collectively, these results suggested ATO sensitized the effects of checkpoint inhibitor in oral and esophageal SCC cancer cells at least partly through degradation of cyclin D1 and upregulation of PD-L1.

## Discussion

Oral and esophageal squamous cell carcinoma (OSCC, ESCC) are among the most common malignant cancers in China and worldwide 34, 35. Recent clinical studies indicated immune checkpoint inhibitors are promising new drugs for treating both OSCC and ESCC 4, 36, 37, and combinatory uses of checkpoint inhibitors with cancer chemotherapy drugs to achieve the better responsiveness is an important area of researches for SCC treatment 38. In this study, we found cyclin D1 is a molecular target for ATO treatment of OSCC and ESCC. Mechanistically, we found ATO induced DNA damage, and nuclear translocation of Cyclin D1 in cancer cells, that are mediated by ROS and Sumoylation of cyclin D1, respectively. Cyclin D1 is known to be involved in DNA damage repair and we found DNA damage induced T286 phosphorylation of cyclin D1, which promotes the proteasomal degradation of cyclin D1 upon ATO treatment of SCC cancer cells.

Cyclin D1-CDK4/6 signaling pathway is frequently altered in SCC cancer cells to promote drug resistance and to maintain the malignant phenotypes of the cancer cells 39. Over expressions of cyclin D1 are correlated with poor clinical outcomes of ESCC and OSCC 40, 41. In this study, we measured cyclin D1 RNA and protein levels in 97 human ESCC tissue samples. We found significant upregulations of the cyclin D1 RNA level in a large percentage of the tissue samples (69%), which is consistent with previous large scale cancer genetic study 31. Interestingly, we observed more frequent upregulations of cyclin D1 RNA levels compared with the protein levels of cyclin D1 by IHC analysis of the human ESCC tissue samples. Previous studies showed Stat1 promotes cyclin D1 degradation in fibrosarcoma cancer cells, and Stat1 is frequently upregulated in ESCC 29, 32. Inversed correlation between p-Stat1 and cyclin D1 was observed in ESCC cancer tissues with IHC staining. We also found increased phosphorylated Stat1 by ATO treatment in ESCC cancer cells. We speculated that upregulations of p-Stat1 in ESCC cancer cells may cause an increase of proteasomal degradation of cyclin D1, resulted in the less dramatic upregulations of protein levels of cyclin D1 in human ESCC tissue samples.

Zhang *et al.* reported cyclin D1-CDK4/6 signaling is an important upstream regulator of PD-L1 degradation, and upregulation of PD-L1 by CDK4/6 inhibition sensitized cancer cells to checkpoint inhibitor therapy 15. In the present study, we showed ATO induced upregulation of PD-L1 through the CDK4/6 -Cul3 pathway both in SCC cancer cell lines and SCC mouse models. Sato *et al.* reported DNA damage induced upregulation of PD-L1 through activation of the JAK1/2-STAT1/2/3-IRF1 signalling pathway 42. Dimco *et al.* reported IFN-γ activated Stat1 directly interacts with Cyclin D1 and promotes its proteasome degradation through its ser-701 phosphorylation site in fibrosarcoma cell line U3A cell line 29. We also observed ATO induced activation of Stat1 mediated by ATO induced DNA damage response. Cyclin D1 degradation is likely to be the key upstream regulatory mechanism for ATO induced PD-L1 upregulation in ESCC and OSCC cells.

Zhao *et al.* reported ATO has the immunosuppressive effect of arsenic trioxide on islet xenotransplantation and they found ATO inhibited proliferation of T lymphocyte and increased the proportion of Foxp3^+^ regulatory T cells in recipient mice 43. Based on our results, we suggest ATO induced upregulation of PD-L1, which negatively regulates T cell immunity, is probably one of the underlying molecular mechanisms of the immune suppressive effects of ATO observed in the mouse islet xenotransplantation model.

Importantly, ATO induced PD-L1 upregulation is just one of the many downstream effectors of CDK4/6 signaling. More systematic analysis indicates CDK4/6 regulate additional downstream signaling programs that confer cellular resistances to immune checkpoint inhibitors 17. Several CDK4/6 inhibitors have shown to be effective in the treatment of a broad variety of solid tumors 44. Our results suggested ATO induced cyclin D1 degradation has a similar effect to CDK4/6 inhibitors, caused inhibition of CDK4/6 signaling, induced cancer cell senescence, and inhibited SCC outgrowth in 4NQO induced OSCC and ESCC mouse models* in vivo*. Additionally, the present study does not preclude some other molecular targets of ATO may also take part in the synthetic effects of co- administration of ATO with checkpoint inhibitor 45. Collectively, our results offered the first preclinical evidences and molecular mechanisms of combinatory usages of ATO and immune checkpoint inhibitors with synthetic effects for cancer treatment that warrant future clinical studies.

## Figures and Tables

**Table 1:** List of primers

	Primers used in cancer tissue experiments	
	Upper primer	Lower primer
Cyclin D1	5'-CCGTCCATGCGGAAGATC	3'-TCACGCTCCTCCTCCAGAAG
GAPDH	5'-GAAGGTGAAGGTCGGAGT	3' -CTTTAGGGTAGTGGTAGAAG

## Abbreviations

ATO: arsenic trioxide
4NQO: 4-nitroquinoline 1-oxide
ESCC: esophageal squamous cell carcinoma
OSCC: oral squamous cell carcinoma
ROS: reactive oxygen species
DCFDA: Dichlorofluorescin diacetate
NK Cell: natural killer cell
APL: acute promyelocytic leukemia
PD1: programmed cell death protein 1
PD-L1: programmed death-ligand 1
HE: haematoxylin and eosin
